# Aleuria Aurantia Lectin (AAL)-Reactive Immunoglobulin G Rapidly Appears in Sera of Animals following Antigen Exposure

**DOI:** 10.1371/journal.pone.0044422

**Published:** 2012-09-14

**Authors:** Songming Chen, Chen Lu, Hongbo Gu, Anand Mehta, Jianwei Li, Patrick B. Romano, David Horn, D. Craig Hooper, Carthene R. Bazemore-Walker, Timothy Block

**Affiliations:** 1 Institute for Hepatitis and Virus Research, Doylestown, Pennsylvania, United States of America; 2 Department of Chemistry, Brown University, Providence, Rhode Island, United States of America; 3 Drexel University College of Medicine, Doylestown, Pennsylvania, United States of America; 4 Departments of Cancer Biology and Neurological Surgery, Thomas Jefferson University, Philadelphia, Pennsylvania, United States of America; Mayo Clinic, United States of America

## Abstract

We have discovered an Aleuria Aurantia Lectin (AAL)-reactive immunoglobulin G (IgG) that naturally occurs in the circulation of rabbits and mice, following immune responses induced by various foreign antigens. AAL can specifically bind to fucose moieties on glycoproteins. However, most serum IgGs are poorly bound by AAL unless they are denatured or treated with glycosidase. In this study, using an immunogen-independent AAL-antibody microarray assay that we developed, we detected AAL-reactive IgG in the sera of all animals that had been immunized 1–2 weeks previously with various immunogens with and without adjuvants and developed immunogen-specific responses. All of these animals subsequently developed immunogen-specific immune responses. The kinetics of the production of AAL-reactive IgG in mice and rabbits were distinct from those of the immunogen-specific IgGs elicited in the same animals: they rose and fell within one to two weeks, and peaked between four to seven days after exposure, while immunogen-specific IgGs continued to rise during the same period. Mass spectrometric profiling of the Fc glycoforms of purified AAL-reactive IgGs indicates that these are mainly comprised of IgGs with core-fucosylated and either mono-or non-galactosylated Fc N-glycan structures. Our results suggest that AAL-reactive IgG could be a previously unrecognized IgG subset that is selectively produced at the onset of a humoral response.

## Introduction

Early detection of exposure to pathogens or toxins is fundamental to medicine and public health [1], [2], but can be challenging when the source and nature of a suspicious agent cannot be readily identified [3]–[5]. This is mainly due to the inability of any detection system to detect exposure to all known and potential pathogens and agents of bioterrorism [2]. In this case, confirmation of exposure to a foreign substance or invading microbe may have utility in prompting intervention. This may be accomplished by detection of an early host response to a toxic exposure, such as onset of the production of target-specific antibodies. At one week after vaccination or infection the immunogen-reactive B cell repertoire is undergoing class-switching and affinity maturation and higher affinity, immunoglobulin-G (IgG) antibodies are beginning to appear in sera [6]. Detection of increasing immunogen-specific antibody titers in sera obtained a number of days apart is generally required to distinguish between acute and existing immune responses but this requires time and identification of the eliciting agent. An approach to confirm that an acute humoral immune response is underway would have therapeutic implications.

IgGs are glycoproteins, normally with a complex N-linked and biantennary glycan, composed of a core heptasaccharide structure with variable addition of fucose and outer arm sugars such as galactose and sialic acids, attached at Asn-297 of the heavy chain C*_H_*2 domain [7], [8]. More than 20 different Fc glycoforms, consisting of the heptasaccharide biantennary core with a combination of different numbers of core-Fucose, Galactose (Gal), bisecting N-Acetyl Glucosamine (GlcNAc), and terminal sialic acids, have been found on polyclonal serum IgGs [9]–[11], as well as a single monoclonal IgG, regardless of their subclass [12], [13]. These glycans play important roles in the structure and function of proteins, such that changes in a single glycan can affect protein folding and processing [14], [15]. Differential glycosylation clearly impacts IgG function. For example, IgG without terminal Galactose (G0 IgG) or core Fucose exhibit higher antibody dependent cell mediated cytotoxicity [7], [16]–[20]. However, the mechanisms involved in the production of different IgG glycoform as well as processes involved in their regulation remain unclear.

Recent studies have shown that the production of specific IgG Fc glycoforms are closely associated with the B cell environment and that certain factors can alter the IgG elaborated glycoforms [9], [21], [22]. Moreover, several diseases have been associated with the abnormal elevation of specific IgG Fc glycoforms. For example, serum levels of G0 IgG are unusually high in rheumatoid arthritis, Myositis Syndromes [23], Lambert-Eaton myasthenic syndrome [24], Crohn's disease, and other inflammatory diseases and are closely correlated with disease severity [25]–[27]. Fucosylated G0 IgG with anti-α-Gal specificity was found to be elevated in patients with liver fibrosis [28]. In mice, G0 IgG levels have been found to rise and then fall back to normal during an immune response [29], while immunogen-specific IgGs in the sera of repeatedly immunized mice have increased fucose content [30]. While all of these observations suggest that IgG with diverse Fc N-glycan structures can be induced under certain immunological or pathological conditions, a systematic study to explore alterations in IgG glycosylation during a conventional immune response has not been done.

Lectin, such as Aleuria Aurantia Lectin (AAL), which specifically binds to exposed core (α-1, 6) and outer arm (α-1, 2 or α-1, 3) linked fucose moieties on different glycans, can be used to assess IgG glycosylation. Although most serum IgGs contain many fucose moieties, they do not bind to AAL in their native state; their fucose moieties must be exposed by either denaturation or digestion with glycosidases for this to occur. However, we have discovered an IgG subset which are naturally produced and are greatly elevated in the serum of people with liver diseases such as cirrhosis, which can bind AAL without denaturation or glycosidase treatment [7]. In this study, we assessed the sera of mice and rabbits over the course of their responses to different immunogens to determine whether AAL-reactive IgGs are produced in a conventional immune response. Lectin-antibody microarray [31]–[34] and mass spectrometry-based IgG Fc N-glycan profiling [9], [23], [24], [35]–[37] were used to assess the AAL binding properties and glycan structure of the serum IgGs, respectively. AAL-reactive IgGs were found to be rapidly induced by immunization, exhibiting kinetics distinct from those of the immunogen-specific response, implying that they are markers of an underlying immune mechanism.

## Results

### Development of a microarray assay for the detection and quantitation of AAL-reactive IgG

**Figure 1:**
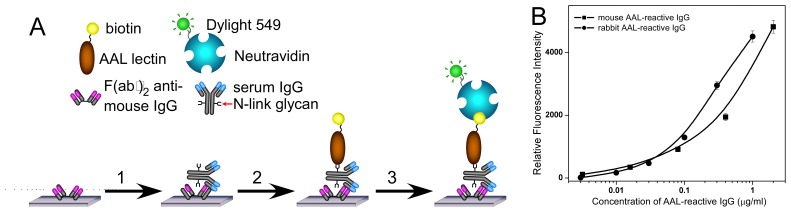
AAL-antibody microarray based AAL-reactive IgG detection assay. (A) A schematic drawing of the AAL-antibody microarray assay for the detection of mouse (or rabbit) AAL-reactive IgG: (1) mouse or rabbit serum samples were incubated on an antibody microarray; (2) AAL lectin was applied on the antibody microarray to detect glycans on the captured IgGs after unbound proteins were washed off; (3) Dylight 549 labeled NeutrAvidin was incubated on the microarray to detect the biotinylated AAL. (B) Representative mouse (▪) and rabbit (•) AAL-reactive IgG standard curves. The standard used for the curve was a completely denatured mouse IgG with known concentration. The capture F(ab′)_2_ was Goat F(ab′)_2_ fragment anti-mouse IgG for mouse IgGs, and Donkey F(ab′)_2_ fragment anti-rabbit IgG for rabbit IgGs, respectively. The relative fluorescence intensity of each data point was subtracted from the blank (PBS control), but were not shown in the curves due to the logarithm X-axis.

A sandwich format, lectin-antibody microarray was developed to detect and quantify AAL reactive IgGs from mouse or rabbit serum samples. In this immunogen-independent assay, F(ab′)_2_ fragments or antibodies specific for mouse and rabbit IgG, or recombinant protein A/G, all of which have very low affinity for AAL, were immobilized on the microarray slide to capture serum IgGs, and biotinylated AAL was used to detect the glycans on the captured IgGs (as shown in Figure 1A). This format allowed us to only measure the glycans on the captured IgGs from serum samples without interference from AAL binding to the capture reagents. To quantify AAL-reactive IgGs, standard curves were prepared in each experiment using denatured mouse and rabbit AAL-reactive IgGs as described in Methods and Materials. These standards were used as denaturation fully exposes fucose moieties in IgG allowing AAL access while naturally occurring AAL-reactive IgGs are rare and not readily obtained from normal sera. Using this AAL-antibody microarray assay, standard curves for mouse and rabbit AAL-reactive IgGs were prepared as shown in Figure 1B. The limits of detection (LOD) are about 10.0 ng/ml in both assays, which is sufficient for the detection of the levels of AAL-reactive IgG normally found in sera. To avoid saturation of the assays, serum was diluted at least 1∶100. This resulted in an actual limit of detection of 1.0 µg/ml for AAL-reactive IgG in serum.. Therefore, 1.0 µg/ml was considered to be the limit of the AAL-reactive IgG concentration in serum detectable in the assay.

### AAL-reactive IgG and immunogen specific IgG elicited by immunization exhibit different kinetics in individual animals

**Figure 2:**
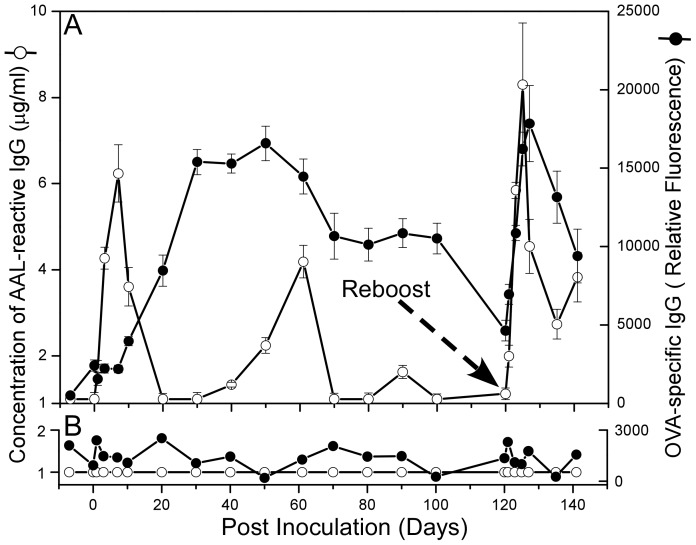
Production kinetics of AAL-reactive and OVA-specific IgG in five OVA/IFA immunized mice and one non-immunized normal mouse measured by using AAL-antibody microarray and normal antibody microarray methods, respectively. The five inoculated mice were reboosted at Day 120. (A) AAL reactive IgG and OVA specific IgG production kinetics of OVA/IFA inoculated mouse. Each point represents the average AAL reactive concentration from 5 inoculated mice calculated according to AAL reactive IgG standard curve. (B) AAL reactive IgG and OVA specific IgG production kinetics of the control (non-immunized) mouse.

To study the production of AAL-reactive IgG in animals, we serially collecting serum samples both prior to (Day −7) and following the inoculation of Balb/c mice with 50 ug of ovalbumin (OVA) in the absence of adjuvant (see Table 1 for the details of all animals studied). Levels of AAL-reactive IgG and OVA-specific IgGs at each time point were assessed by AAL-reactive IgG microarray. As expected due to the poor immunogenicity of OVA in the absence of adjuvant, neither OVA specific IgG nor AAL-reactive IgG were detected in any mice. Serially-obtained sera from a second group of Balb/c mice immunized with 50 ug OVA in incomplete Freund's adjuvant (OVA/IFA) and then boosted with OVA/IFA 120 days later were similarly assessed (Figure 2). As shown in Figure 2B, a control, non-immunized mouse produced neither detectable AAL-reactive IgG nor OVA specific IgGs at any time during the four-month monitoring period. On the other hand both AAL-reactive IgG and OVA specific IgG were detected in OVA/IFA inoculated mice (as shown in Figure 2A). Notably, the kinetics of the appearance of AAL-reactive IgG and OVA-specific IgG are distinct. The OVA-specific IgG response exhibited a typical IgG development curve, rising consistently from several days after immunization to a peak at Day 50, then decreasing to levels close to baseline before rapidly rising following the Day 120 boost. In contrast, AAL-reactive IgG appeared quickly, rising to a peak at Day 5 after immunization rapidly disappearing by day 20, then spontaneously reappearing at high levels approximately 2 months and lower levels 3 months after immunization. Like OVA-specific IgG, AAL-reactive IgG levels rapidly increased following booster immunization.

**Table 1:** Animals, immunogens and routes of immunogen administration that were used in the mice and rabbit immunization studies.

Animal	Group	Species	Gender	Number of animals	Animal ID	Immunogen	Dosage per animal	Routes
Mouse	1	Balb/c	Female	5	N/A	OVA/IFA	100 µg	IP
Mouse	2	Balb/c	Female	5	N/A	KLH	100 µg	IP
Mouse	3	Balb/c	Female	1	N/A	N/a	N/A	N/A
Mouse	1	C57/BL6	Female	5	N/A	OVA/CFA	100 µg	IP
Mouse	2	C57/BL6	Female	5	N/A	RV (108ffu)	100 µl	IP
Rabbit	1	NZW	Male	2	Rabbit #2Rabbit #3	OVA/CFA	500 µg	subQ
Rabbit	2	NZW	Male	2	Rabbit #4Rabbit #5	KIN/CFA	500 µg	subQ
Rabbit	3	NZW	Male	1	Rabbit #1	N/A	N/A	N/A

Abbreviations: OVA: ovalbumin; KLH: keyhole limpet hemocyanin; CFA: complete Freund's adjuvant; IFA: incomplete Freund's adjuvant; IP: Intraperitoneal injection; subQ: Subcutaneous Injection; NZW: New Zealand White; RV: rabies virus (uv inactivated); KIN: Kininogen.

### AAL-reactive IgG is elicited during immune responses to various stimuli in both mice and rabbits

**Figure 3:**
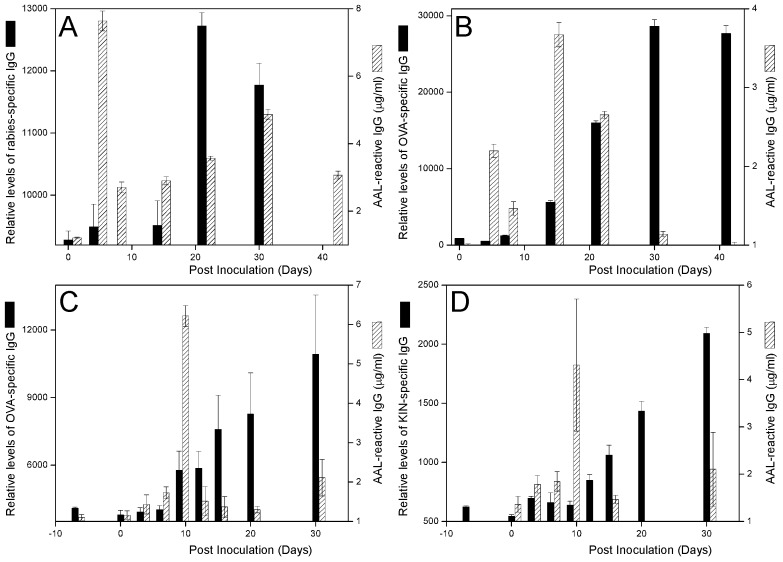
Production kinetics of AAL-reactive IgG and immunogen specific IgG production in immunized mice and rabbits measured by using AAL-antibody microarrays. Different animals were immunized with different immunogens as shown in Table 1. Both immunogen specific IgG and AAL-reactive IgG were measured in the serum samples from each animal. The black bar in the graphs showed averaged immunogen-specific IgG level (relative levels according to the fluorescence intensities); and the strip bars in the graphs showed averaged AAL-reactive IgG levels. (A) Five mice (strain C57BL/6) were inoculated with inactivated rabies virus; (B) Five mice (strain C57BL/6) were inoculated with OVA/CFA; (C) Two rabbits were inoculated with OVA/CFA, (D) Two rabbits were inoculated with KIN/CFA.

To determine whether or not AAL-reactive IgG may be a more universal indicator of the onset of a humoral immune response, we inoculated different mouse strains and rabbits with a variety of immunogens via different routes as summarized in Table 1. Serum AAL-reactive IgG and immunogen specific IgG levels were measured using AAL-reactive IgG microarrays and antigen-specific assays as described in Methods and Materials. As shown for C57BL/6 mice inoculated with RV (Figure 3A), C57BL/6 mice inoculated with OVA/CFA (Figure 3B), rabbits inoculated with OVA/CFA (Figure 3C), and rabbits inoculated with KIN/CFA (Figure 3D) the rapid rise-and-fall kinetics of AAL-reactive IgG is a characteristic feature of the onset of an immune response. In each case the immunogen-specific IgG response developed considerably later than that of AAL-reactive IgG, with peak responses occurring weeks later. Neither AAL-reactive IgG nor immunogen-specific IgG was detected in control animals that were not inoculated.

### AAL-reactive IgG has low affinity for the immunogen that elicited its production

**Figure 4:**
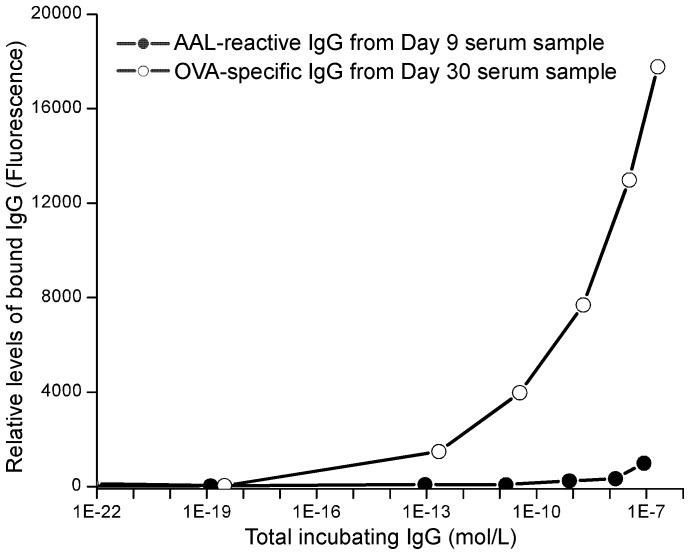
Comparison of OVA-binding affinities of AAL-reactive IgG and OVA-specific IgG at peak levels. AAL-reactive IgG and OVA specific IgG were purified from OVA/CAF inoculated rabbit by using OVA Agarose and AAL Agarose beads immunoprecipitation as described in Methods and Materials. The AAL-reactive IgG was purified from Day 9 serum sample, and the OVA-specific IgG was purred from Day 30 serum sample of rabbit 2 (as shown in Table 1), respectively. The purified serum samples were serially diluted and applied onto a protein/antibody microarray that immobilized OVA protein and donkey F(ab′)_2_ anti-rabbit IgG spots. The bound IgG levels on OVA protein, and the concentration of IgG were measured simultaneously. The OVA-bound AAL-reactive IgG and OVA-specific IgG were plotted as a function of their concentration measured by the immobilized donkey F(ab′)_2_ anti-rabbit IgG.

To establish whether or not the AAL-reactive IgG appearing early in the immune response is specific for the immunizing antigen we assessed the OVA binding affinity of rabbit AAL-reactive IgG elicited by OVA/CFA immunization (Rabbit 2 in Table 1) using a protein/antibody microarray. Serum samples collected at Day 9 after inoculation for AAL-reactive IgG and 11 days later for OVA specific IgG were used to assess affinity for OVA. Immunoprecipitation (IP), as described in Methods and Materials was used to isolate AAL-reactive and OVA-specific antibodies. Binding affinities for OVA were measured using a sandwich protein/antibody microarray in which anti-rabbit IgG F(ab′)_2_ and pure OVA protein (Sigma Aldrich) were used as the immobilized, capture reagents. This format allowed us to measure concentrations of both total IgG (anti-IgG spots) and OVA-specific IgG in single samples (OVA protein spots). The OVA binding curves of purified AAL-reactive IgG and OVA specific IgG were plotted with the relative fluorescence intensity of the OVA protein spot presented as a function of the fluorescent intensity of the total anti-IgG spot. As shown in Figure 4, due to the concentrations of the purified antibodies neither curve reached a plateau. However, based on the differences between the curves we estimate that the apparent Kd value of AAL-reactive IgG for OVA is at least 100 times lower than that of the OVA-specific antibody.

### Mass spectrometric glycoform profiling reveals that AAL-reactive IgG has core-fucosylated and under-galactosylated Fc N-glycan structures

**Figure 5:**
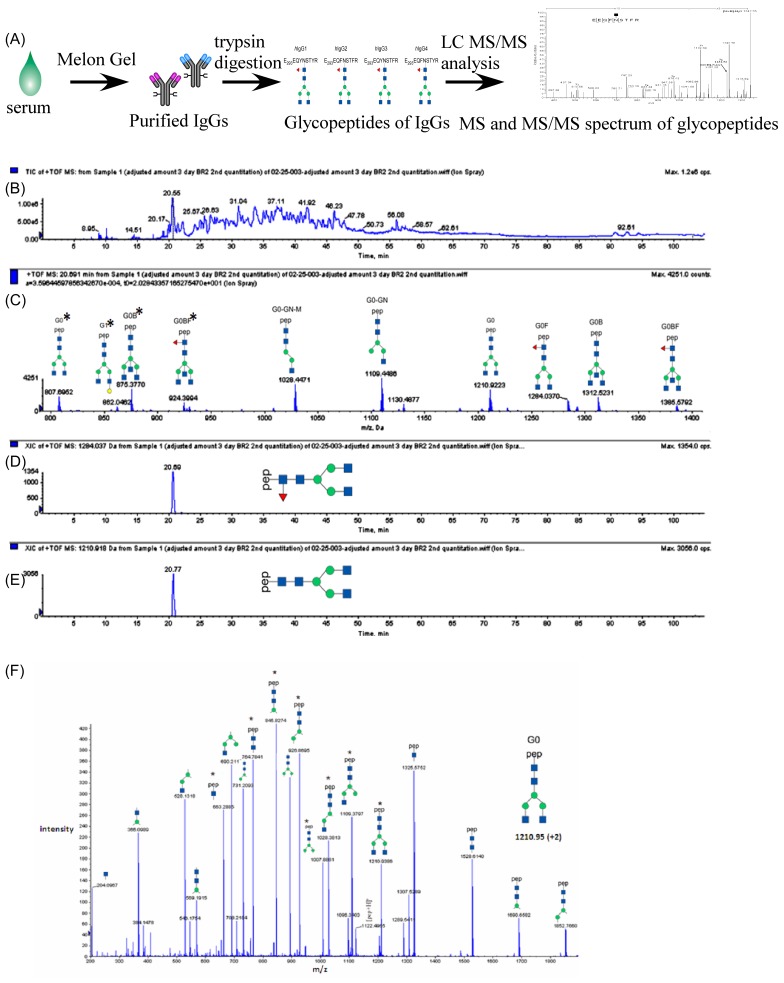
Use mass spectrometric identification and glycoform profiling of the IgG glycoforms and AAL-reactive IgG. (A) Scheme of the procedure (see details in Experiment and Methods Section); (B) A total ion chromatograph of a purified rabbit IgG; (C) A mass spectrum of the sample at retention time 19 minutes; (D) and (E) Extracted ion chromatographs of G0F, and G0, respectively. (F) MS/MS spectrum of G0 IgG.

While the specificity of AAL for fucosylated proteins or oligosaccharides [38], indicates that AAL-reactive IgG is fucosylated, AAL-affinity based analysis cannot provide details of the N-glycan structure of AAL-reactive IgG. Therefore, we used a mass spectrometry-based approach to provide further insight into the glycan structures of AAL-reactive IgG. Our method takes advantage of the fact that there is only a single N-linked glycosylation site at Asn 297 of the C*_H_*2 domain of antibody, and that a 9-mer glycopeptide fragment that includes both Asn 297 and glycan from trypsin-digested IgG can be directly analyzed on a mass spectrometer. As depicted in Figure 5 and described in Methods and Materials, the Asn 297 and N-glycan containing peptide can be selected by the mass spectrometer, and its glycan structure identified by MS/MS spectra. The levels of each identified glycopeptide, twice the molar concentration of the parent IgG glycoforms, are then determined in extracted ion chromatograms. Since the amount of mouse serum samples that we collected were insufficient for glycoform profiling using mass spectrometry, we purified AAL-reactive IgG from KIN/CFA immunized Rabbit 4 (Table 1) using AAL-Agarose immunoprecipitation followed by Melon gel treatment as described in Methods and Materials. A serum sample that was incubated with “empty” Agarose beads (Pierce) was used as a control. The purified AAL-reactive IgG and control samples were first denatured, trypsin digested, and then injected into a nano flow LC MS/MS (ABI Q-Star Élite). Glycopeptides of IgGs always elute off the reverse phase column at the beginning of the LC gradient as shown in Figure 5B. For quantification and kinetic profiling of each IgG Fc-glycoform, mass spectrometry analysis was set at MS mode, in which only the precursor ions were scanned (Figure 5C). The extracted ion chromatogram (XIC) for each glycoform was plotted such that the relative abundance for each is represented by its peak area. Examples of XICs for G0F and G0 are shown in Figures 5D and 5E respectively. To determine the glycan structure of each glycopeptide, MS/MS analysis was performed on each XIC peak. Figure 5F shows a representative MS/MS spectrum for the G0 glycopeptide.

A total of 22 different IgG glycoforms were identified in rabbit IgG samples using mass spectrometric glycoform profiling described in Methods and Materials. Prior to immunization, nine of these glycoforms, consisting of 18.8% of the total IgG, were Fc-fucosylated. Only 11 glycoforms were detected in purified, AAL-reactive IgGs, with 84.5% being core-fucosylated, and the rest non-fucosylated. Moreover, AAL-reactive IgG primarily consist of under galactosylated forms, in which 45.9% are fucosylated mono-galactosylated (G1F), fucosylated mono-galactosylated with bisecting GlcNAc (G1BF), or fucosylated mono-galactosylated lacking one GlcNAc (G1F-GlcNAc), and 39.4% are fucosylated agalactosyed (G0F), fucosylated agalactosylated lacking one GlcNAc (G0F-GlcNAc), and fucosylated agalactosylated with bisecting GlcNAc (G0BF). G2 species, such as fucosylated di-galalctosylated (G2F) or fucosylated di-galactosylated with bisecting GlcNAc (G2BF) were not found in purified AAL-reactive IgG. Fucosylated glycoforms in AAL-reactive IgG were 3.5 times more abundant than those in normal rabbit IgG.

### Kinetics of the appearance of AAL-reactive and core-fucosylated, under galactosylated IgG are correlated

**Figure 6:**
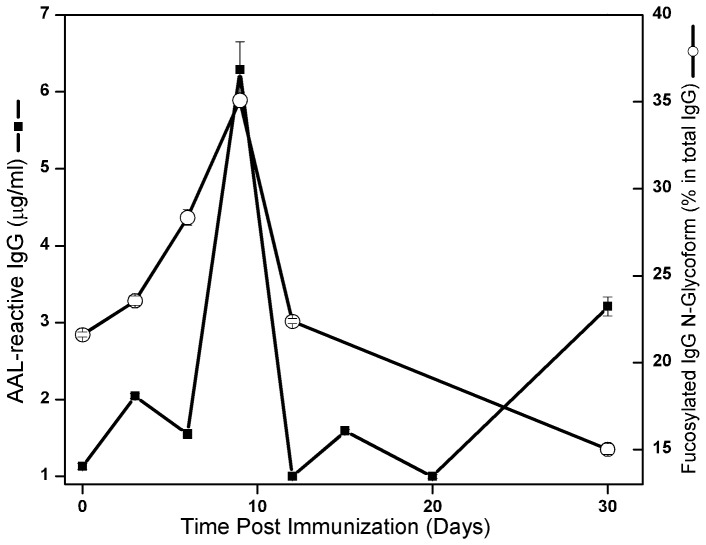
Kinetics curves of fucosylated IgGs (solid line with open circles (○) corresponding to the right Y-axis), which were measured by using mass spectrometry, and AAL-reactive IgGs (solid line with solid squares (▪) corresponding to the left Y-axis), which were measured by using AAL antibody microarray, in serum samples from rabbit 4 which was inoculated with KIN/CFA. The total fucosylated serum IgG levels were the sum of the percentage of each fucosylated glycoform (as shown in table 2, AAL-reactive IgG column) at each time point. The AAL-reactive IgG levels were measured by using AAL-antibody microarray methods as described in Methods and Materials.

To further probe the relationship between AAL-reactivity and IgG glycoforms, we used mass spectrometry to quantify the glycoforms of total serum IgG at each time point following immunization of Rabbit 4 with KIN/CFA (Table 2) and plotted the results against the levels of AAL-reactive IgG determined by microarray. While the concentration of total IgG remained constant (not shown in the figure), changes in the total level of all fucosylated glycoforms as well as that of AAL-reactive IgGs showed similar kinetics, both rapidly rising to peak approximately 10 days after immunization then quickly falling (Figure 6).

**Table 2:** Fc glycoforms of total IgG purified from Day 0 serum sample and AAL-reactive IgG purified from Day 9 sample of OVA-inoculated rabbit (rabbit# 4) were identified and quantified by using mass spectrometry method.

No	Name	Glycan composition	2+ m/z	3+ m/z	Content in non immunized rabbit (%)	Content in AAL IP purified IgG (%)
1	G1F	H4N4F1	1365.05	910.37	3.4	22.7
2	G1BF	H4N5F1	1466.5	978	1.8	13.8
3	G0F	H3N4F1	1284.03	856.35	6.2	12.2
4	G0F-GlcNAc	H3N3F1	1182.5	788.67	1.9	11.8
5	G0BF	H3N5F1	1385.56	924.04	3.2	11.7
6	G2	H5N4	1373.05	915.7	1	7.3
7	G1F-GlcNAc	H4N3F1	1263.5	842.67	0.5	6.2
8	G1B	H4N5	1393.5	929.33	3.1	6.1
9	G0F-GlcNAc-Man	H2N3F1	1101.5	734.67	0.8	3.7
10	G1FNeu5Gc	H4N4F1S1	1518.63	1012.75	0.4	3.2
11	G2Neu5Gc	H5N4S1	1474.55	983.45	0.5	0.7
12	G2FNeu5Gc	H5N4F1S1	1547.67	1032.11	0.3	0.7
13	G0-2GlcNAc-Man	H2N2	926.9	618.27	1.4	0
14	G0-2GlcNAc	H3N2	1007.9	672.27	1.3	0
15	G0-GlcNAc-Man	H2N3	1028.45	685.97	8.8	0
16	G0-GlcNAc	H3N3	1109.43	739.95	16.3	0
17	G1-GlcNAc	H4N3	1190.48	793.99	2.3	0
18	G0	H3N4	1210.95	807.63	20	0
19	G0B	H3N5	1312.52	875.35	16.2	0
20	G1	H4N4	1291.94	861.63	9.5	0
21	G2F	H5N4F1	1446.08	964.39	0.4	0
22	G1Neu5Gc	H4N4S1	1445.58	964.05	1.4	0
Total Fucosylated Glyco Isoforms					18.8	84.5
Total non-fucosylated glycol isoforms					81.2	15.5

## Discussion

**Figure 7:**
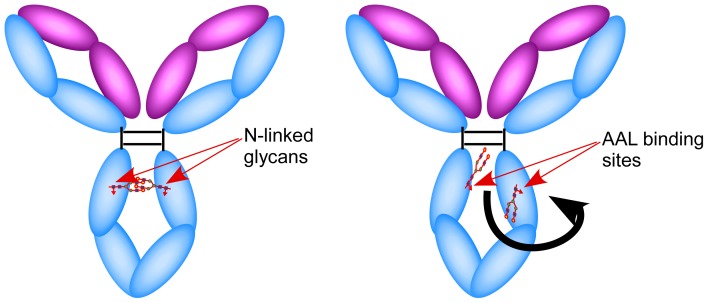
A hypothetical model of non AAL-reactive IgG in “closed” (*left*) and a “flip-out or open” (*right*) conformation. The “closed” conformation may not allow AAL access to the fucose moieties on IgG Fc-glycan, but the “open” conformation might.

To our knowledge, this is the first report of the induction by an immune response of an IgG that, in its native form, is bound by the fucose-selective lectin AAL. The Fc N-glycan of IgG is commonly core fucosylated with over 30% of total mouse or human IgGs being fucosylated G0 IgG. However, under normal circumstances IgG is poorly reactive with AAL. The Fc N-glycan of IgG is located in the C*_H_*2 domain close to the hinge region and, importantly, the conformation of C*_H_*2 is stabilized through glycan-glycan interactions between the two heavy chains. Thus, when IgG is in its normal conformation the core-fucose is buried within the cleft between the two Fc heavy chains and is inaccessible to AAL lectin (Figure 7 *left*). Consequently a change in IgG glycosylation or quaternary structure would be required for the core-fucosylated Fc N-glycan structure to become accessible to AAL, for example, alterations resulting in “open” or “flip-out” conformations [39] exposing AAL-reactive fucose moieties (Figure 7 *right*). A recent study using hydrogen-deuterium exchange suggests that glycosylation alteration of Fc N-glycan can change conformation of C*_H_*2 domain, suggesting this “open” or “flip-out” conformation possibly exists [40]. Galactose moieties in the glycan-glycan interactions between the two IgG heavy chains may make important contributions to IgG conformational stability as enzymatic removal of galactose residues makes IgG AAL-reactive [17], [31], [36], [39]. Based on these observations we expect that the AAL-reactive IgG detected in the current study is a fucosylated and under galactosylated IgG that possesses an “open” conformation.. However, we cannot predict as to whether differences in the IgG amino acid sequence contribute to the formation of an AAL-reactive structure.

Although the AAL-reactive IgGs described here are a natural product of the immune response, it is unclear whether they are properly folded or misfolded. Since denatured IgGs are also AAL-reactive and do not bind to their target antigen, it is possible that the immunogen-non-specific AAL-reactive IgGs detected in this study are misfolded byproducts of IgG production. However, the mass spectrometry data, which measures AAL-reactive IgG by mass instead of binding affinity, indicates that these IgGs contain more fucose than conventional IgG (Figure 6). Furthermore, AAL-reactive IgG bound efficiently to immobilize protein A/G suggesting that A/G binding site, located in the C*_H_*2 domain, may be intact. These observations tend to suggest that the AAL-reactive IgG described here is a novel class of IgG that is transiently produced during an immune response and not simply a denatured or miss-folded IgG molecule. However, it is conceivable that the newly produced AAL-reactive IgG could be locally misfolded, at only the N-glycan region of the C*_H_*2 domain. Further experiments are needed to determine whether or not this may be the case.

The rise-and-fall kinetics of the production of AAL-reactive IgG is distinct from that of the immunogen-specific IgG produced in the same animal. Two additional “waves” of AAL-reactive IgG were observed following the initial peak detected shortly after immunization. While the reason for these “waves” is unknown it is interesting to speculate that they may have some relationship to stages in the antigen-specific response to the immunogen. Notably, each successive wave of AAL-reactive IgG production after primary immunization was reduced while the response following antigen boost had the characteristics of a conventional recall response. Outside of the rapid appearance of AAL-reactive IgG in the sera at the onset of an immune response, the significance of this kinetic pattern is unknown.

Due to its lack of specificity for the inducing antigen, AAL-reactive IgG is unlikely to contribute to the immune response. Nevertheless, we noted a general association between the magnitude of the peak AAL-reactive IgG response and that of the antigen-specific response which occurred considerably later. Further experiments are required to confirm whether or not the level of the AAL-binding IgG may be an early predictor of the magnitude of the specific response to an immunogen.

The distinct structure, lack of antigen specificity and unique production kinetics of AAL-reactive IgG lead us to question their genesis. There are several potential cell sources including conventional B cells, either naïve or memory, or B-1 B cells, that are either non-specifically stimulated by cytokines or rapidly lose antigen specificity. B-1 B cells appear unlikely since they do not develop memory which is a characteristic of the AAL-reactive IgG response indicated by a more rapid onset and higher levels of serum AAL-reactive IgG at boost. On the other hand, IgGs with different glycan structures are produced by maturing conventional B cells [9]. However as opposed to being the direct products of B cells, AAL-reactive IgG could be serum IgG that has been recycled by another cell type such as dendritic cells, macrophages and endothelial cells. This alternative hypothesis is supported by the dynamic changes in different IgG glycoforms during AAL-reactive IgG production. In the mass spectrometric glycoform profiling of total IgG from a KIN/CFA immunized rabbit, greater than 20% of the agalactosylated (G0) IgG present in serum disappeared during the time that AAL-reactive IgG was produced (see Figure S1). Thus, AAL-reactive IgG could be converted G0 IgG. If this is the case, the rapid decrease in AAL-reactive IgG could result from either a shorter half-life or recycling back into the circulation as G0 IgG.

Our results suggest that the appearance of AAL-reactive IgG may be a common event during the early stages of an immune response. If so, AAL-reactive IgG may serve as a universal biomarker for the early detection of an immune response to agents including pathogens and toxins, even when their identity is unknown. The production of AAL-reactive IgG may allow discrimination between an acute and prior immune response. Currently, raising antibody titers in consecutive serum samples is taken as reasonable evidence of an ongoing response, a process that can delay diagnosis. The presence of AAL-reactive IgG would raise the possibility that any concurrently detected response may be acute and should be considered for treatment. The more rapid commencement of treatment based on the presumption of an acute host response could have a large impact in disease control and anti-bioterrism applications. Importantly, our animal immunization results suggest that the appearance of AAL-reactive IgG in sera is related to a specific antigen challenge rather than day-to-day environmental antigen exposure. The observation that OVA-specific IgG and AAL-reactive IgG were both elicited by OVA in the context of adjuvant, while neither was detected in mice given OVA alone, suggests that AAL-reactive IgG is a selective biomarker for the onset of humoral immunity.

## Materials and Methods

### Animal care and immunization

All animal studies were carried out in strict accordance with the recommendations in the Guide for the Care and Use of Laboratory Animals of the U.S. Public Health Services/National Institutes of Health. All studies using Balb/c mice and white rabbits were carried out according to protocols that were approved by the Committee on the Ethics of Animal Experiments of Lampire Biological Laboratories (IAUF#: G0-IgG Kinetics IAUF, protocol# IHVR-DOD-01-2009 for mice study; and IAUF#: Core Rabbits Freunds IAUF, protocol# IHVR-DOD-01-2011). These animals were housed in the animal facilities of Lampire Biological laboratories, and all immunizations, blood taken, and serum preparation were done in the facility according to Standard Operation Procedure of Lampire Biological Laboratories. All studies using C57BL/6 mice were carried out in accordance with Public Health Service Policy on Humane Care and Use of Laboratory Animals under protocols approved by the Institutional Animal Care and Use Committee of Thomas Jefferson University (Animal Welfare Assurance Number A3085-01). The mice were housed in the animal facilities at TJU, and all immunizations, blood taken, and serum preparation were done by Dr Li according to Standard Operation Procedure of Dr Hooper's lab and TJU.

As shown in Table 1, two strains of mouse, Balb/c and C57BL/6 mice, and New Zealand White rabbits were used in the study. Ovalbumin (OVA), Kininogen (Low Molecular Weight), and inactivated rabies virus were used to inoculate the animals through IP or sub Q. Blood was drawn by using either orbital bleeding (on mice) or vein (rabbits) at different time points before and after immunization. The general frequency of blood taken was between 3 to 5 days during the first month of post immunization and reboost, then 7 to 10 days intervals after the first month. Pre-immunization blood was taken 7 days prior to immunization done at Lampire, but at the same day of immunization done at TJU. The actual time points could vary 1 to 2 days due to staff schedule changes or holidays. One mouse or rabbit, which was bred in the same conditions but received no injection, was used as control animals for the experiments done in Lampire. Day 0 serum samples of C57BL/b mice were used as controls for the experiments done in TJU.

### Lectin and antibodies

Wild type Lectin AAL was purchased from Vector labs. Recombinant AAL was constructed and expressed with 10× His tag in E coli, and purified with Ni-NTA beads (Qiagene) [41]. All anti-mouse or rabbit IgG antibodies, including both whole IgG, and F(ab′)_2_ fragments, were purchased from Jackson ImmunoResearch Laboratories, Inc.

### Antibody microarray preparation and Quantification of AAL-reactive IgG by using AAL-antibody microarray assays

High density antibody microarray was prepared for the detection of antigen specific IgG and AAL-reactive IgG as described previously [42]–[46]. Before microarray fabrication, the 1“X3” ultrathin nitrocellulose coated glass slides (PATH, Gentel Biosciences, Wisconsin) were separated into 52 identical rectangular areas (4 columns by 12 rows) by wax grids imprinted by using a wax imprinter (Gel Company, CA). For the selection of an optimal capture antibody for AAL-reactive IgG detection, different anti-mouse, or rabbit antibodies or F(ab′)_2_ fragments were printed onto each of the 52 identical subbarrays on each PATH slide by using a Scienion FLEX ARRAYER S3 ultra low volume piezo microarrayer. About 300 pico liters of antibody was printed and resulted in a high density spot at the diameter of 130 micron. For mouse AAL-reactive IgG detection, we used goat F(ab′)_2_ anti-mouse IgG as the capture antibody; for rabbit AAL-reactive IgG, we used donkey F(ab′)_2_ anti-rabbit IgG as the capture antibody.

To quantify AAL-reactive IgG, the printed antibody microarray slides were pre-equilibrated to room temperature and blocked with 1% IgG-free BSA (Lampire, PA) in 10 mM phosphate saline buffer, pH 7.2 with 0.5% Tween 20 (PBST0.5) for one hour at room temperature. After the slides were rinsed with PBST0.5 after the incubation and then 6 µl of 100 times diluted serum samples in PBST0.1 were applied onto the different subarrays in a random order. After the slides were rinsed with PBST0.1 three times for 3 minutes, biotinylated AAL was applied onto each subarray to probe AAL-reactive IgGs. After rinse with PBST0.1, the slide was probed with Dylight 549 labeled streptavidin (Pierce) and scanned by using a Perkin Elmer ScanArray Lite microarray scanner at resolution of 10 micron. The images were analyzed and the data was extracted by using Genepix software (Molecular Device, CA).

To prepare mouse and rabbit AAL-reactive IgG standards, 1.0 mg/ml of pure mouse or rabbit IgGs (Jackson ImmunoResearch Laboratories) was incubated at 50°C in 20 mM DTT in PBS buffer for 2 hours. The reaction mixtures were desalted by using Biospin P-6 columns that were pre-equilibrated with PBS. The completely denatured IgGs (AAL-reactive IgG standard) were serially diluted to concentrations from 10 µg/ml to 1 ng/ml in PBS buffer. The serially diluted samples were applied onto different subarrays of the microarray described above accompanied with other serum samples for AAL-reactive IgG detection. The AAL binding intensity of standards and unknown serum samples were extracted by using software as described above. Standard curves of AAL-reactive IgG were plotted, and AAL-reactive IgG concentration in unknown samples were calculated.

### Quantify immunogen-specific IgGs by using antibody microarrays

In order to measure the relative level Immunogen-specific IgGs, we immobilized 0.5 mg/ml of immunogen in PBS (Kininogen, Ovalbumin, or rabies virus) on each subarray of the microarray slides containing 52 identical subarrays. The microarrays were blocked by using 1% IgG free Bovine Serum Albumin (BSA) (Lampire Biologicals Inc.) in PBST0.5, and then were washed with PBST0.5. Serum samples from animals were diluted 100 times by using PBST0.1, which were applied onto each subarrays and incubated for 1 hour at room temperature. The captured anti-immunogen antibodies were then probed by using biotinylated anti-mouse IgG (for mouse serum samples), or anti-rabbit IgG (for rabbit serum samples). Dylight 549 labeled streptavidin (Pierce) was finally applied onto each subarray to probe biotinylated anti-IgGs. After the microarray slides were scanned on the ScanArray Lite, the fluorescence intensity from each spot was extracted from the microarray images by using GenePix Pro 6.0 software.

### Isolation of AAL-reactive IgG and immunogen-specific IgGs by using Immunoprecipitation

Immunoprecipitation (IP) of AAL-reactive IgG was done by using homemade recombinant AAL Agarose beads; and IP of OVA or KIN specific IgG was done by using homemade OVA or KIN Agarose beads. AAL, OVA, or KIN -Agarose beads were prepared by incubating recombinant AAL, OVA, or KIN protein with NHS-activated Agarose beads (Pierce) according to the manufacture instructions. After conjugation, the AAL, OVA or KIN beads were washed withPBST0.1, and then blocked with 1% BSA in PBST0.1 for 1 hour at room temperature. In order to isolate AAL-reactive IgG, OVA-specific IgG, or KIN specific IgGs from serum samples, AAL-, OVA- or KIN-Agarose beads were incubated with rabbit serum samples at 4°C overnight with gently shaking. The identical serum samples were incubated with the empty control Agarose beads (Pierce) at same conditions. AAL-reactive IgG was eluted by washing the AAL-Agarose beads with PBS containing 150 mM of fucose. The AAL IP eluent was further purified by using Melon Gel (Pierce) according to the manufacturer's instructions. OVA and KIN specific IgGs were eluted by washing the OVA- and KIN Agarose beads with Gly-HCl pH3.0 buffer, respectively. The eluted OVA-, or KIN- specific IgGs was immediately neutralized by using 1 M Tris solution.

### Determine antigen binding affinity of purified AAL-reactive IgG using antibody microarrays

To determine the antigen binding affinity of purified immunogen-specific IgGs and AAL-reactive IgG, pure immunogen-antigen (such as OVA) and anti-rabbit F(ab′)_2_ were immobilized onto a protein/antibody microarray. Serial diluents of purified immunogen specific IgG and AAL-reactive IgG were applied onto microarray, followed by probing with biotinylated goat anti-rabbit IgG. The microarray was then probed with Dylight 549-labeled NeutrAvidin. The relative fluorescence intensities from bound IgGs were read out from the protein (OVA) spots. The concentration of the AAL-reactive IgG was calculated according to a standard curved of serial diluted AAL-reactive IgG standards. Titration curves were plotted by using the concentrations of the bound immunogen specific IgG or AAL-reactive IgG as a function of the concentration of incubated immunogen specific IgG or AAL-reactive IgG. The 50% of effective concentration (EC50), which was considered as the apparent disassociation constant (Kd), was obtained as a estimated Kd of the immunogen-specific IgG or AAL-reactive IgG binding to the immunogen (OVA).

### IgG Fc N-glycoform profiling by using mass spectrometry

Quantification of IgG glycol isoforms with mass spectrometry was carried out as described previously [8], the procedure was shown in Figure 5A. Briefly, purified rabbit IgGs from serum samples at different days of pre- or post- immunization were denatured and modified by using DTT and 2-Iodoacetamide, followed by trypsin digestion in ammonium bicarbonate buffer overnight at room temperature. Tryptic peptides were de-salted using homemade C_18_ capillary column, and then re-dissolved in solvent A (2%ACN, 0.1% formic acid). LC-MS and LC-MS/MS analysis was performed on a QSTAR Elite QTOF mass spectrometer (Applied Biosystems, Foster City, CA) equipped with a Tempo nano LC system. Samples were loaded onto a pre-column (75 um×3 cm) packed with 5 um Monitor C_18_ particles (Column Engineering. Ontario, CA) and then eluted by a linear gradient from 0% to 80% solvent B (98% ACN, 0.1% formic acid) over 80 minutes on a homemade analytical column (75 um×10 cm of 3 µm C_18_ Monitor particle) with 3 µm ID tip. The ionization voltage was set at 2 kV. Precursor ions were scanned over the mass range from 500–1800 and MS/MS spectra were acquired for selected ions under automatic collision energy. Ions for glycopeptides were also imported into the inclusion list for fragmentation using fix collision energy ramp from 36 to 44. Quantitative analysis of each glycoform was done at MS mode only. Intensity of each subtype was obtained by adding the peak area of +2 and +3 forms of the same glycopeptide. The glycopeptide sequence of rabbit IgG, E_248_QQFNSTIR_256_ (GenBank#: AAA64252.1) was used for the identification and quantitation of glycopeptide and its glycoforms. This was also the only glycopeptide sequence we found in GenBank, other possible subclasses were not found either in the database or in our mass spectrometry data analysis.

## Supporting Information

Figure S1 Comparison of kinetics of G0 and fucosylated IgG Fc N-glycoforms of rabbit that was immunized with kininogen in Complete Freund's Adjuvants (KIN/CFA) (see rabbit 4 in Table 1). The content of glycoform (Y-axis) represents the percentage of these two glycoforms in total IgGs. G0 glycoforms (solid black line with solid square symbols) is the sum of G0, bisecting G0 (G0B), and G0 lack of one GlcNAc (G0-GlcNAc); the fucosylated glycoform (solid red line with solid round symbols) represents the sum of all the fucosylated glycoforms in Table 2. Percentage of each glycoform in total IgG was measured and calculated by using mass spectrometric glycoform profiling as described in Methods and Materials section. Each data point is the average of three repeated measurements of the same samples. This result shows that content of G0 glycoforms has an oppose trend during the first two weeks of immunization: it decreased when the fucosylated glycoforms increased, and increased when fucosylated glycoform decreased.
(PDF)
