# Supplementary material for: Aleuria Aurantia Lectin (AAL)-Reactive Immunoglobulin G Rapidly Appears in Sera of Animals following Antigen Exposure
Source: PLoS One. 2012 Sep 14;7(9):e44422. doi: 10.1371/journal.pone.0044422 (PMC3443102; doi:10.1371/journal.pone.0044422)
Supplement: Figure S1 — Comparison of kinetics of G0 and fucosylated IgG Fc N-glycoforms of rabbit that was immunized with kininogen in Complete Freund's Adjuvants (KIN/CFA) (see rabbit 4 in Table 1). The content of glycoform (Y-axis) represents the percentage of these two glycoforms in total IgGs. G0 glycoforms (solid black line with solid square symbols) is the sum of G0, bisecting G0 (G0B), and G0 lack of one GlcNAc (G0-GlcNAc); the fucosylated glycoform (solid red line with solid round symbols) represents the sum of all the fucosylated glycoforms in Table 2. Percentage of each glycoform in total IgG was measured and calculated by using mass spectrometric glycoform profiling as described in Methods and Materials section. Each data point is the average of three repeated measurements of the same samples. This result shows that content of G0 glycoforms has an oppose trend during the first two weeks of immunization: it decreased when the fucosylated glycoforms increased, and increased when fucosylated glycoform decreased. (PDF) [file pone.0044422.s001.pdf]

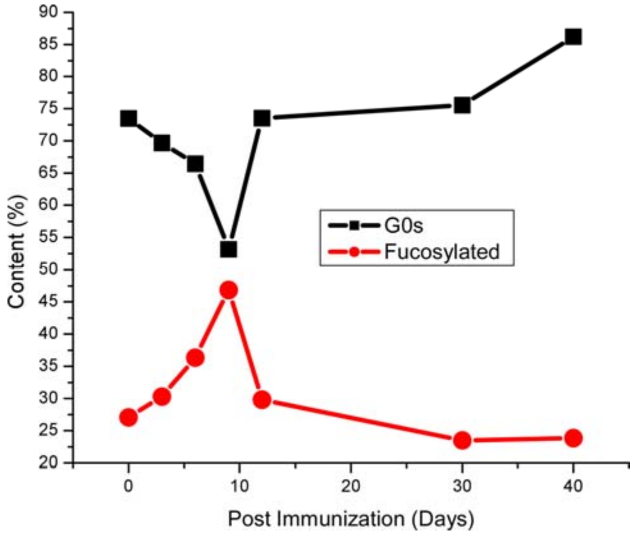

Figure S1 Comparison of kinetics of G0 and fucosylated IgG Fc N-glycoforms of rabbit that was immunized with kininogen in Complete Freund's Adjuvants (KIN/CFA) (see rabbit 4 in Table 1). The content of glycoform (Y-axis) represents the percentage of these two glycoforms in total IgGs. G0 glycoforms (solid black line with solid square symbols) is the sum of G0, bisecting G0 (G0B), and G0 lack of one GlcNAc (G0-GlcNAc); the fucosylated glycoform (solid red line with solid round symbols)represents the sum of all the fucosylated glycoforms in Table 2. Percentage of each glycoform in total IgG was measured and calculated by using mass spectrometric glycoform profiling as described in Methods and Materials section. Each data point is the average of three repeated measurements of the same samples. This result shows that content of G0 glycoforms has an oppose trend during the first two weeks of immunization: it decreased when the fucosylated glycoforms increased, and increased when fucosylated glycoform decreased.
